# MHC Multimer-Guided and Cell Culture-Independent Isolation of Functional T Cell Receptors from Single Cells Facilitates TCR Identification for Immunotherapy

**DOI:** 10.1371/journal.pone.0061384

**Published:** 2013-04-26

**Authors:** Georg Dössinger, Mario Bunse, Jeannette Bet, Julia Albrecht, Paulina J. Paszkiewicz, Bianca Weißbrich, Isabell Schiedewitz, Lynette Henkel, Matthias Schiemann, Michael Neuenhahn, Wolfgang Uckert, Dirk H. Busch

**Affiliations:** 1 Institute for Medical Microbiology, Immunology and Hygiene, Technische Universität München, Munich, Germany; 2 Focus Group “Clinical Cell Processing and Purification”, Institute for Advanced Study, Technische Universität München, Munich, Germany; 3 Clinical Cooperation Groups ‘‘Antigen-specific Immunotherapy’’ and “Immune Monitoring”, Helmholtz Center Munich (Neuherberg) and Technische Universität München, Munich, Germany; 4 DZIF - National Centre for Infection Research, Munich, Germany; 5 Max-Delbrück-Center for Molecular Medicine, Berlin, Germany; 6 Humboldt-Universität, Berlin, Germany; University of Colorado Denver, United States of America

## Abstract

Adoptive therapy using T cells redirected to target tumor- or infection-associated antigens is a promising strategy that has curative potential and broad applicability. In order to accelerate the screening process for suitable antigen-specific T cell receptors (TCRs), we developed a new approach circumventing conventional *in vitro* expansion-based strategies. Direct isolation of paired full-length TCR sequences from non-expanded antigen-specific T cells was achieved by the establishment of a highly sensitive PCR-based T cell receptor single cell analysis method (TCR-SCAN). Using MHC multimer-labeled and single cell-sorted HCMV-specific T cells we demonstrate a high efficacy (approximately 25%) and target specificity of TCR-SCAN receptor identification. In combination with MHC-multimer based pre-enrichment steps, we were able to isolate TCRs specific for the oncogenes Her2/neu and WT1 even from very small populations (original precursor frequencies of down to 0.00005% of CD3^+^ T cells) without any cell culture step involved. Genetic re-expression of isolated receptors demonstrates their functionality and target specificity. We believe that this new strategy of TCR identification may provide broad access to specific TCRs for therapeutically relevant T cell epitopes.

## Introduction

Transgenic expression of antigen-specific TCRs has gained relevance through clinical trials indicating that specific breakup of tolerance towards tumor-associated auto-antigens can be achieved by reinfusion of *ex vivo*-isolated, gene-modified, autologous, patient-derived T cells. Further clinical data indicate that elevated TCR binding strength, as a means to elicit site-directed auto-antigen recognition, can result in enhanced immunogenicity [1]–[3]. It is generally believed that central tolerance limits the prevalence of high-avidity TCRs specific for auto-antigens within the autologous peripheral T cell repertoire. However, there is evidence that a substantial number of self-reactive TCRs are recruited into regulatory T cell compartments, which might be difficult to access with current cell culture based approaches [4], [5].

Furthermore, the allo-repertoire has been proposed as an alternative source of therapeutically efficient TCRs, most convincingly demonstrated by complete leukemia remissions upon allogeneic donor lymphocyte infusions (DLIs) [6]. A large retrospective study of 4643 stem cell transplantations revealed a pronounced correlation between defined HLA-mismatches and reduced leukemia relapse. This finding indicates that T cells may provide better protection if their cognate epitopes are presented by mismatched HLAs rather than matched HLAs [7]. An explanation for this finding might be the absence of thymic presentation of mismatch-HLA presented epitopes, which in principle permits the occurrence of high avidity T cells that recognize tumor-associated auto-antigens. This assumption is further supported by the finding that high-avidity T cells from the T cell repertoire of HLA-mismatched donors can be isolated by *in vitro* expansion of T cell clones [8]–[10].

However, since not all T cells are expandable under similar conditions, *in vitro* culture-based protocols limit access to restricted TCR repertoire compositions [11], [12]. This limitation could best be overcome by direct, *ex vivo* single-cell sorting of antigen-specific T cells and subsequent TCR cloning from individual cells, without the need for any *in vitro* propagation. In principle, this could be achieved by combining MHC multimer-staining [13] with single-cell TCR sequencing.

Although many epitope-specific T cell populations are extremely rare they can be accurately detected through the combination of MHC multimer-based pre-enrichment and combinatorial MHC multimer staining technologies [14], [15]. However, it has not yet been possible to combine MHC multimer staining with single-cell TCR identification, since the simultaneous extraction of both chains of the hetero-dimeric receptor is technically highly challenging. Several single-cell-based TCR sequencing efforts have been described, most using sets of degenerate primers binding to consensus motifs [16]–[21] or rapid amplification of cDNA ends (RACE) PCR [22], [23].

Although these strategies resulted in single-cell-derived TCR sequences, it has not been shown that correct pairing to functionally reconstruct the receptor of the original cell can be obtained, e.g. by transgenic re-expression of the identified TCR chains. There are several technical concerns that require careful interpretation of sequencing results without further analysis of the obtained receptor. For example, in the case of consensus primer-based approaches, V-segment domains are truncated outside the primer binding sites; since single nucleotide polymorphisms (SNPs) within V-segments have been described to influence ligand binding, reliable sequence reconstruction might be limited [15], [24]. Another threat that must be carefully controlled is potential cross-contamination between samples, as highly sensitive nested PCR amplifications are prone to contamination and require extensive protective measures as well as sufficient numbers of controls. During the establishment of our TCR-SCAN approach, we indeed had to deal with contaminants that - without the necessary precautions and permanently implemented in-process controls - easily could have been misinterpreted as belonging to a common repertoire present in different donors.

With this report we provide first proof-of-concept evidence that by combining a novel single-cell sequencing approach and MHC multimer selection procedures, fully functional TCRs can be extracted from even extremely rare antigen-specific T cell populations. This method will give direct and unbiased access to antigen-specific TCRs independent of T cell differentiation status or function. We believe that this strategy has broad applicability and will advance the field of adoptive T cell therapy.

## Results

### Development of a TCRα/β-specific RACE-PCR with high efficacy at the single-cell level

**Figure 1:**
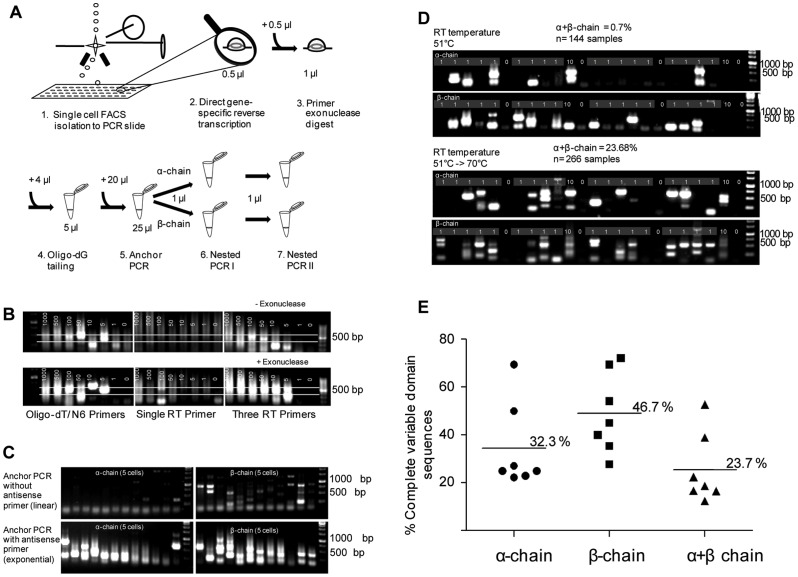
Optimized RACE-PCR-based approach for TCR sequencing from single cells. (A) Sketch of the basic principle of the novel single-TCR sequencing strategy. Reverse transcription and exonuclease digest were consecutively done on-slide. Complete reactions were transferred to 96 well plates for tailing, anchor PCR and nested PCR round I and II. Alpha and beta chains were pre-amplified together during anchor PCR and in separate reactions for nested PCRs I and II. To change the reaction conditions for the first four steps of the protocol, the volume was increased to create optimal conditions for the subsequent enzyme. For nested PCR round I and II 1 µl of the previous reation volume was transferred to the next step. (B) Different priming strategies (standard oligo-dT/random hexamers, one gene-specific primer and three gene-specific primers per TCR chain) were tested with and without extra exonuclease-I digest of residual RT-primers. Decreasing numbers of human T cells, from 1000 to 1 as indicated served as template. (C)The anchor PCR step is used to prolong the oligo-dG overhang from the tailing step. PCR was tested without reverse primer in “linear” mode and with reverse primer in “exponential” mode of amplification. (D) Temperature switch during reverse transcription was tested. RT at a constant temperature of 51°C (upper row) was compared with a temperature increase form 51°C for 30 min to 70°C for 20 min (bottom row). (E) In seven independent single-cell PCR experiments a total number of 266 samples were processed. Numbers of samples yielding α- and/or β- chain products above the evaluated full-length cut-off size were calculated as a percentage of total samples per experiment. Mean values for all experiments taken together are indicated by horizontal lines.

We first followed a recently described nested RACE-PCR-based approach for TCR sequence identification [22]. However, in our hands this protocol was not sensitive enough for efficient access to single cells, which forced us to introduce extensive protocol modifications. We ended up with a procedure as depicted in Figure 1A. This scheme gives an overview of the entire method. The different steps are described in more detail later in this section. Single-cell samples were isolated by FACS-sorting on a PCR glass wafer from Advalytix, and reverse transcription (RT) and exonuclease-I digest was directly performed on the slide before the samples were transferred to 96-well plates to perform the following steps. We modified the resulting cDNA transcripts by enzymatically adding a stretch of oligo-dG to the 3′ end. This step generated an artificial priming site upstream of the unknown V-segment sequence (Fig. S1). We prolonged this artificial priming site by an additional PCR-step using a primer that binds to the oligo-dG stretch and has a 51 bp long overhang. We further refer to this step as anchor PCR.

Pfu-polymerase based enzymes have been shown to digest primers that bind DNA sequences over a part of their sequence but not the 3′ end. The single stranded 3′ end can be degraded leaving a truncated but completely annealed primer. It was shown that this exonuclease activity can be blocked by coupling the nucleotides on the 3′ end of a primer over a phosphorothioate (PTO) group [163]. The primer dC-Anchor PTO (Table S1) contains a 51 bp long overhanging 5′ sequence and otherwise consists only of cytosine bases, which makes false priming very likely. Therefore, to prevent the elongation of unwanted priming events the 3′-terminal nucleotide of this primer was coupled over PTO. Two sequential nested PCRs utilizing the resulting artificial priming site were added to increase sensitivity and specificity. Beginning with a reaction volume of 0.5 µl for RT we scaled up the volume to create optimal reaction conditions for the respective enzymes in the consecutive steps.

### Optimization of different procedures to increase PCR sensitivity

As shown in Figure. 1B, major improvements in sensitivity were achieved by using a set of three gene-specific RT primers, each for α- and β-chain (TCRAC1/2/3 and TCRBC1/2/3), instead of commonly used standard oligo-dT primers, random hexamer primers (N6) or a single gene-specific primer. To determine the sensitivity limit of our protocol we used decreasing cell numbers, from 1000 cells to a single cell, as a template. we tested different priming strategies with this experimental approach. Besides standard oligo-dT RT, we applied two previously published primers [22] and – each, for α respectively β-chain - three step-like arranged primers (TCRAC1/2/3 and TCRBC1/2/3). This assay yielded no clear band, probably because non-specific PCR interactions increased along with escalating amounts of non-specific nucleic acids and other cellular components. For illustration see Figure S1.

We reasoned that residual RT primers, as a potential substrate for the tailing enzyme, could become the main source of nonsense products in the following PCR steps. Thus, in line with a previous report, we confirmed that exonuclease-I digest of residual RT primers reduced the degree of non-specific priming in serial PCR amplifications [25]. TCR cDNA at this point is still bound to the mRNA template and is therefore protected against exonuclease activity [25]. By combining exonuclease digest of residual RT primers and use of several serial RT-primers we achieved amplification of a dense PCR product smear down to five cells.

Nevertheless, at this point the method seemed not sensitive enough to amplify the TCR from a single cell (data not shown). To determine, whether the product amplification by the PCR steps was limiting for the sensitivity we tried to increase the number of amplification steps. The anchor PCR step initially served to prolong the artificial priming site; for this reason a single forward primer was used, resulting in linear product amplification. However, reverse priming turned this step into an exponential amplification, which resulted in a striking improvement of sensitivity (Figure 1C). A schematic overview of the entire priming strategy is provided in Figure S1. Although we could obtain PCR products from less cells with this modification single cell samples still failed to yield a detectable product.

To further refine sensitivity, we again focused on the RT reaction, as conversion of the minute amounts of easily degradable mRNA seemed to be a very delicate step in our protocol. We carefully determined the optimal RT temperature by testing a range of temperatures between 40°C and 70°C (Figure S2). With this strategy we succeeded to isolate the TCR from single cells, however with insufficient efficiency (Fig. 1D). We suspected that the RT step was most critical, as mRNA is relatively instable. For this reason we tried to further optimize the RT temperature. Temperature reduction to 51°C had already slightly improved the sensitivity, but the most prominent improvement was observed upon addition of a short-term extension at 70°C, despite a rapid loss of enzyme activity at this temperature (Figure 1D).

Without these important modifications, less than 1% of single-cell samples resulted in full-length TCR α- and β-chains, whereas, with the temperature increase from 51°C to 70°C the, approx. 25% of all single-cell samples contained full-length products of the complete variable part of TCR sequences, including the start codon. In some cases, several differently sized bands were detected within one sample. Sequencing of all the bands usually gave sequence information for the same TCR chain, whereby shorter bands represented fragments that were truncated in the V-segment. Samples resulting in PCR bands ranging in size between 500 bp and 750 bp were confirmed to contain complete sequences. For a more detailed method evaluation, 266 samples from three different donors were tested in seven independent experiments. For α-chains, the recovery rates for individual experiments ranged between 20% and 75%, for full-length β chains between 25% and 78% and for both chains between 10% and 50% of all single-cell samples (Figure 1E). More detailed informations about these experiments are provided in the following figures.

### Determination of efficacy and specificity of single-cell TCR extraction targeting CMV-specific T cell populations

**Figure 2:**
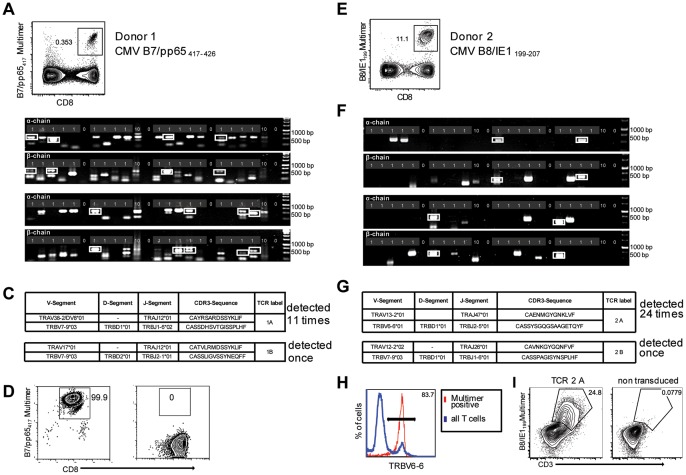
Single-cell PCR delivers sequences of functional CMV-specific TCRs. (A) PBMCs from Donor 1 were recovered and stained with HLA-B7/pp65_417–426_multimers. Dot plot shows the further analyzed CD8 and MHC multimer double-positive cell population. Cells were pre-gated on live lymphocytes (propidium iodide-negative, and CD3-positive). (B) TCR SCAN as described in Figure 1 and agarose gel electrophoresis of the resulting PCR products was performed. The photography shows the agarose gel. Upper row shows α-chain products matched with the respective β-chains in the lower row. White boxes indicate α- and matched β-chain-products derived from identical single cell samples. (C) The table summarizes the V- D- J- segment type and amino acid sequences of TCRs identified from CMV-multimer positive T cells in Figure 2A. TCR1A was identified 11 times and TCR 1B was detected once. (D) MHC multimer-positive T cells from the same donor were *in vitro* expanded and six T cell clones were successfully maintained. All clones contained TCR1A as confirmed by PCR and sequencing. The left FACS plot shows HLA-B7/pp65_417–426_ staining and the right FACS plot shows staining with an irrelevant MHC multimer. (E) PBMCs from donor 2 were recovered and stained with HLA-B8/IE-1_199–207_ multimers. The dot plot shows the further analyzed CD8 and MHC multimer double-positive cell population. Cells were pre-gated on living lymphocytes (propidium iodide negative and CD3 positive). (F) TCR SCAN as described in Figure 1 and agarose gel electrophoresis of the resulting PCR products was performed. Upper row shows α-chain products matched with the respective β-chains in the lower row. White boxes indicate α- and matched β-chain-products derived from identical single cell samples. (G) The table summarizes the V- D- J- segment type and amino acid sequences of TCRs identified from CMV-multimer positive T cells in Figure 2E. The respective nucleic acid sequences of all TCR rearrangements all are summarized in Table S2 and S3. TCR 2A was detected 24 times; TCR 2B only once. (H) PBMCs from Donor 2 were labeled with MHC multimer and antibody binding to V-segment of TCR 2A Vβ6-6. Red line shows MHC multimer-positive cells. Blue line shows total T cells. (I) The identified TCR sequence from TCR 2A was expressed in Jurkat76 T cells by retroviral gene transfer. Transduced (left FACS plot) and non-transduced (right FACS plot) Jurkat76 T cells were analyzed for expression of CD3 and MHC multimer binding.

In order to further validate TCR-SCAN, we FACS-isolated (under ‘highest purity mode’) single cells from a CMV-specific T cell population by labeling with HLA-B7/pp65_417–426_ multimers (Figure 2A). For each experiment one PCR slide was loaded with 36 single-cell samples. Four samples, each with 10 cells served as positive controls. To control for potential cross contamination during the entire process, eight positions were left empty to serve as negative controls. The PCR protocol (Figure 1A) was started within 48 h after cell isolation, and PCR products were detected by agarose gel electrophoresis. For sequencing, the PCR products were gel extracted and subsequently cloned into a carrier plasmid.

Samples yielding both α-and β-chain in full lengths are highlighted by white boxes (Figure 2B). Within the analyzed T cell population, we repeatedly detected the same TCR α/β- pairing (Figure 2C); only once a different sequence was identified, indicating clonal dominance. We aligned all DNA-sequences of the TCR that we repetitively isolated and found them to be 100% identical, which is expected in an antigen-specific T cell population that is specific for a chronic pathogen (data not shown). To confirm this interpretation and to exclude cross contamination, we isolated T cells from the same donor and expanded clones by limiting dilution. We obtained six T cell clones, and PCR analysis exclusively identified the same TCR α/β-pairing as detected in the single-cell PCR-derived samples. The TCR sequences of the clones were 100% identical to the single-cell-derived TCR sequences and MHC multimer staining (Figure 2D).

The second TCR α/β-pairing sequenced from this CMV-reactive T cell population has been described previously, and the TCR was identified as part of the public repertoire [26]. These data confirm that by TCR-SCAN, functional, antigen-specific TCR sequences can be extracted at single-cell resolution.

To demonstrate that complete functional TCR isolation from single cells is adaptable to T cells with other epitope specificities, we analyzed CMV-specific T cells from a different donor. T cells were labeled with HLA-B8/IE-1_199–207_ multimers and underwent single-cell sorting (Figure. 2E). Subsequent TCR-SCAN extraction yielded full-length TCR α/β-pairings in 10% of all isolated single cells (Figure 2F).

To our surprise, sequencing again revealed mostly identical TCR α/β-chains among the analyzed samples. The experiment was repeated to exclude cross contamination and to obtain larger sample numbers. In total we obtained the sequence of the dominant TCR in 24 single-cell derived samples (Figure 2G), and a different TCR α/β-combination was observed in only one sample. The respective nucleic acid sequences of all TCR rearrangements all are summarized in Table S2 and S3. Again, strong clonal dominance characterized the large HLA-B8/IE-1_199–207_-specific T cell population. To obtain further evidence for the presence of a highly dominant T cell clonotype, we analyzed the original T cell population for its content of Vβ6-positive cells. As shown in Figure 2H, more than 80% of all MHC multimer-positive T cells expressed the TRBV6-6*01 V-segment, which was also detected by TCR-SCAN (Figure 2G).

### Proof of the antigen specificity of a single cell derived TCR by gene transfer

To confirm antigen specificity of the TCR sequence isolated on the single-cell level, we cloned the dominating full-length TCR α- and β- chain sequences isolated on the single-cell level, we cloned the dominating full-length TCR α- and β- chain sequences into the retroviral expression vector pMP71 and transduced the human T cell lymphoma line Jurkat76. This cell line is deficient in an endogenous TCR, and therefore CD3 only appears on the cell surface upon successful transgenic expression of a TCR. Moreover, stable transgenic expression of CD8α co-receptor renders Jurkat76 cells suitable for MHC class I multimer labeling. As shown in Figure 2I, upon transduction a substantial portion of the cells could be stained with MHC multimers.

These data formally prove that MHC multimer-guided single-cell sorting and subsequent single-cell TCR extraction identifies antigen-specific and functional TCR sequences.

### Direct TCR sequence isolation from a very small and diverse antigen-specific T cell population

**Figure 3:**
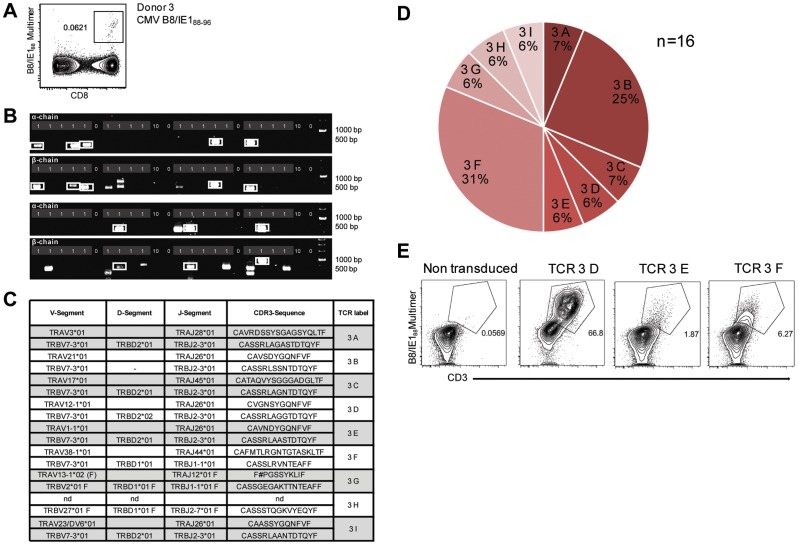
Characterization of a small diverse T cell repertoire and transgenic expression of detected TCRs. (A) PBMCs from donor 3 were stained with HLA-B8/IE-1_88–96_ multimers. Dot plot shows the further analyzed CD8 and MHC multimer double-positive cell population. Cells were pre-gated on living lymphocytes (propidium iodide-negative and CD3-positive). (B) A PCR slide with single antigen-specific T cells from Figure 3A were FACS-isolated. TCR SCAN as described in Figure 1 and agarose gel electrophoresis of the resulting PCR products was performed. Upper row shows α-chain products matched with the respective β-chains in the lower row. White boxes indicate samples α- and matched β-chain-products derived from identical single cell samples. (C) The table summarizes the V- D- J- segment type and amino acid sequences of TCRs identified from CMV-multimer positive T cells in Figure 3A. In three independent experiments we identified nine different TCRs (TCR 3A-I) (D) Pie chart indicates the prevalence of identified TCRs from donor 3. Percentages represent incidence of respective TCR divided by total number of positive samples. (E) Sequences from TCR 3D, 3E and 3G were expressed in Jurkat76 T cells by retroviral gene transfer. Non-transduced (left FACS plot) and transduced Jurkat76 T cells were analyzed for expression of CD3 and MHC multimer binding.

Both CMV-specific T cell populations analyzed above by TCR-SCAN (Figures 2A and 2E) were quite large and therefore easily detectable by MHC multimer staining. In addition, they were extremely oligoclonal, with one dominating TCR sequence. In such situations, the dominant TCR sequence might also be extracted from the population using less sensitive *in vitro* expansion or direct TCR cloning-based methods. Therefore, we next analyzed a very small CMV-specific population with a frequency of less than 0.1% of all CD3^+^ T cells (Figure 3A). Peripheral blood mononuclear cells (PBMCs) were stained with HLA-B8/IE-1_88–96_ multimers, and single antigen-specific T cells were isolated by FACS (Figure 3A). TCR-SCAN yielded paired full-length TCR α/β-sequences in 20% of all analyzed samples (Figure 3B). The assay was repeated twice in completely independent experiments (blood samples were even taken from the donor on separate days); full-length TCR identification efficacy was very similar and identical TCR α/β-pairings were identified in both experiments. We determined full-length sequences for nine different TCRs. For one TCR, only a non-productive α- (TCR 3G) rearrangements and for one (TCR 3H) only the β-chain were detected (Figure 3C).

In total we obtained sequence information from 16 single T cells and could confirm the pairing of several TCR α/β-combinations in several samples (Figure 3D). The respective nucleic acid sequences of all TCR rearrangements all are summarized in Table S2 and S3. Clonal dominance as found in the previously analyzed CMV-specific T cell populations was not detected. However, a high degree of similarity within Vβ usage and CDR3 regions of different TCRs was found. CDR3 amino acid sequences separated for α- and β-chain were subjected to multiple sequence alignment. The highest degree of sequence similarity was found in the pairings B,D,E and I. Here α-chain CDR3 domains uniformly consisted of 12 amino acids, and the associated β-chain CDR3 domains consisted of 15 amino acids. More than 66% and 80% of all positions were identical for the α-chains and β-chains, respectively. The grade of resemblance considering similar chemical properties among different amino acids for both chains was even 100% (Figure S3).

In order to prove that the obtained sequences indeed translate to productive T cell receptors, we selected three TCR sequences, cloned them into retroviral expression vectors and demonstrated epitope-specific multimer-binding on transduced Jurkat76 cells (Figure 3E). In summary, these data show that combining MHC multimer staining with TCR-SCAN can characterize diverse TCR repertoires even within very small T cell populations.

### Functional characterization of a tumor-antigen-specific TCR isolated ex vivo from an extremely rare population

Encouraged by the finding that even very small antigen-specific T cell populations can be used for culture-independent identification of functional TCR sequences, we asked whether this new technology might also provide access to extremely rare TCR sequences with high clinical relevance.

As source for the identification of high-affinity TCR sequences with specificity for tumor-associated self-antigens, allogeneic donor lymphocytes (with a mismatch in the target-relevant HLA restriction) have been explored. In this setting no negative selection against the target epitope and the associated MHC-molecule takes places, which in principle enables the prevalence of high avidity T cells. Despite the presence of a substantial fraction of alloreactive T cells within allogeneic donor lymphocytes, defined epitope-specific T cells are extremely rare, as indicated by enumerations deduced from *in vitro* cell culture protocols. Two recent reports could show that – at least in the mouse – it is possible to visualize rare epitope-specific naïve precursor cells by a combination of MHC multimer pre-enrichment and combinatorial MHC multimer staining [14], [15]. Based on these observations, we hypothesized that it should be possible to derive tumor antigen-specific TCR sequences from allogeneic donor lymphocytes with MHC-mismatched MHC multimers, independent of *in vitro* cell propagation.

**Figure 4:**
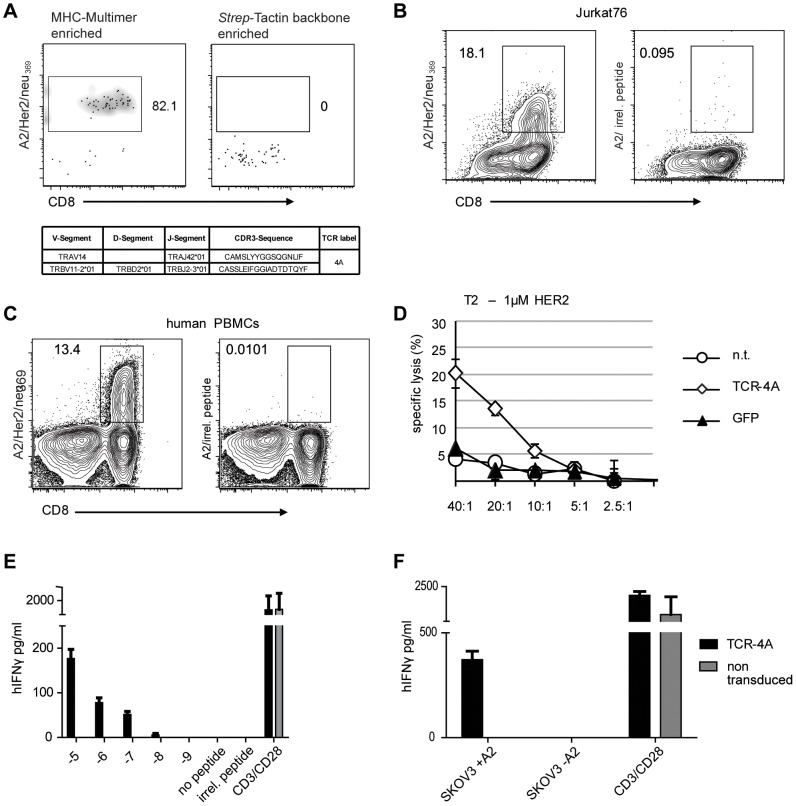
Characterization of functionality of a TCR isolated from rare antigen specific T cells. (A) Cells double-positive for HLA-A2/Her2neu_369–377_ PE- and BV421-conjugated multimers were FACS sorted. The left FACS plot shows CD8 and MHC multimer staining of the cell fraction enriched with Her2/neu specific MHC-multimer. The right FACS plot serves as a negative control that was stained in the same way, but for MACS enrichment only Strep-Tactin backbone was used. Ten single-cell samples were prepared and processed for TCR sequencing. Full-length variable domain sequences of α- and β-chain full-length variable domain sequences from one specific TCR was identified and is shown in the table. (B) Sequence from TCR 4A was expressed in Jurkat76 T cells by retroviral gene transfer. Left FACS plot shows A2/Her2/neu_369–377_ staining and right FACS plot shows staining with an irrelevant MHC multimer. (C) Sequence from TCR 4A was expressed in human PBMCs by retroviral gene transfer. Left FACS plot shows A2/Her2/neu_369–377_ staining and right FACS plot shows staining with an irrelevant MHC multimer. (D) For functional testing 2×10^3^ peptide-loaded T2 cells (1 µM) labeled with ^51^Cr were incubated for 4 h with TCR-4A modified PBLs at different effector/target ratios (E/T ratios). After 4 h free ^51^chrome was quantified in the supernatant. TCR-specificity was controlled by co-incubation with GFP-transduced and non transduced (n.t.) PBMCs. All conditions were tested in duplicates. (E) For detection of peptide specific release 1×10^5^ T2-cells were pulsed with an equal number of Her2/neu peptide titrated from 10^−5^–10^−9^ M concentration. Equal numbers of transduced (black bar) and non-transduced (grey bar) T cells were co-incubated for 4 hours and IFNγ concentration in the supernatant was measured by ELISA. As positive control T cells were stimulated with CD3/CD28 and as control for peptide specificity T2-cells were pulsed with 1 µM of an irrelevant peptide. IFNγ concentration in the supernatant was measured by ELISA. (F) For detection of tumor specific activity10^5^ tumor cells and equal numbers of transduced (black bar) and non transduced (grey bar) human PBMCs were co-incubated for 4 hours and IFNγ concentration in the supernatant was measured by ELISA. As positive control T cells were stimulated with CD3/CD28. All conditions were tested in triplicates.

We performed magnetic pre-enrichment of MHC multimer-binding cells and subsequently labeled the antigen-enriched cell fraction with a differently labeled HLA-A2/Her2neu_369–377_ multimer (fluorophore BV421) as published [15]. We performed TCR-SCAN and were able to isolate a paired full length sequence of one TCR. Figure 4A shows the FACS staining of the enriched and double MHC-multimer stained cell fraction as well as the sequence information we obtained. In order to demonstrate that this particular TCR sequence is really specific for the selected Her2/neu_369–377_ epitope, we performed retroviral transfer into Jurkat76 cells. Most impressively, A2/Her2/neu_369–377_ multimer staining of TCR-transduced cells confirms that we were indeed able to extract a functional TCR from extremely rare precursor frequencies of epitope-specific T cells by TCR-SCAN (Figure 4B). For further functional testing, we retrovirally transduced the TCR into human PBMCs and first demonstrated antigen specificity by the acquisition of MHC-multimer staining (Figure 4C). To show that this specific ligand binding translates to adequate TCR signaling, we pulsed T2 cells with 1 µM specific Her2/neu peptide and co-incubated PBMCs transduced with the newly identified TCR to measure specific killing of target cells and IFNγ secretion (Figures 4D,E). Even when competing for cell surface expression with an endogenous TCR up to 20% of all target cells were lysed in a peptide-specific manner and secretion of IFNγ was detectable down to a peptide concentration of 10^−9^ µM. To finally demonstrate that this TCR would also recognize real tumor cells we used the ovarian cancer cell line SKOV-3, which expresses Her2/neu. This cell line usually is negative for HLA-A2 and thus cannot present the epitope in question. A HLA-A2 transgenic version was used to test for IFN-γ secretion in response to the naturally processed and presented epitope, non-transduced A2 negative cells served as a control for specificity. After co-incubation of 1×10^5^ tranduced PBMCs and with an equal number of the respective tumor cells for 24 h IFN-γ secretion was measured by ELISA. The selective detection of IFN-γ in the A2 transgenic sample demonstrates that TCR-4A is able to functionally respond to Her2-expressing tumor cells in an MHC and epitope specific manner.

To test whether our TCR isolation strategy works for a different auto-antigen, we repeated MHC-multimer enrichment with a WT1-specific MHC-multimer (Fig S4A). To our surprise the enrichment yielded 495 cells, which is far more than previously detected for Her2/neu. We sorted 36 single cells and performed TCR-SCAN. As previously described for the CMV-specific T cell populations none of the eight negative controls showed a PCR product. Interestingly we detected the same TCR pairing (TCR5B) several times. The complete full-length α- and β-chain-pairs shown in Figure S4B were sequenced three times. Beside these two sequences, we repeatedly identified an additional non-productive β-chain in the same sample (data not shown). Some samples with only α- or β-chain similarly yielded one of those three chains. Altogether, three samples contained the matched full-length α- and β-chain of TCR5B and five more samples yielded one or two of the three chains. Apart from TCR5B only one different pairing was found (TCR5A). Again, to confirm antigen-specificity we transduced TCR5A and TCR5B to human PBMCs by retroviral transfer. MHC multimer staining of the transduced cells demonstrated a high transduction efficiency and specificity (Fig. S4C).

## Discussion

In this report we present a novel strategy for straightforward identification of full-length sequences of antigen-specific T cell receptors with specificity for potentially any therapeutically relevant T cell epitope. TCR identification at the single-cell level has multiple advantages over currently available methods. During *in vitro* as well as *in vivo* expansion, the TCR repertoire is shaped towards defined characteristics, which might limit access to the therapeutically most effective TCR sequences. For example, it has recently been reported that chronic infection can lead to dominant expansion of low-avidity T cell populations, which might be less effective for immunotherapy [27]. As chronic antigen stimulation by tumor tissue creates a similar situation for tumor-infiltrating lymphocytes, *in vivo* TCR repertoire changes might limit successful identification of most effective TCR sequences for immunotherapy. Therefore, the analysis of naïve T cell repertoires for therapeutic TCR sequences that have not been shaped by chronic exposition to antigen might be more successful, since a broad variety of different TCR sequences with diverse antigen recognition capacities can be expected. TCR-SCAN allows identification of complete V-segment sequences; therefore, for transgenic re-expression no reconstruction of variable TCR parts is needed, which can be complicated by the presence of individual and functionally relevant SNPs within V-regions. With the protocol presented in this report, the output of our strategy could be further improved by accelerating the downstream processing of PCR products. Especially in combination with next generation sequencing, this method might be used for large-scale identification of T cell receptors pre-defined for antigen-specificity by MHC multimer labeling. TCR-SCAN could serve to build up a large library of TCR sequences potentially useful for clinical application.

The frequency of antigen-specific T cells in the naïve murine T cell repertoire has been determined in earlier reports [14], [15]. However, the clonal composition of these cells has never been explored; such insights should become accessible by our novel approach. Large amounts of sequence information on TCRs recognizing the same epitope could help to further elucidate common rules of binding properties. In earlier reports, the allo-repertoire was suggested as a promising source of TCRs with high avidity [8], [9], [28]. With our novel strategy we can detect antigen-specific T cells in the allogeneic repertoire (Figure 4) as well as in the autologous repertoire (Figure S4). As a potential source for clinically relevant T cell receptors with high avidity for self-derived tumor antigens, we will explore the regulatory T cell compartment in more detail. Less prevalent HLA-types should also be analyzed, further broadening the applicability of adoptive T cell receptor gene therapy. A limitation of our strategy might be the dependence on MHC multimer reagents for T cell identification. However, many of the most prevalent HLA class-I types have already successfully been used for the generation of MHC multimers. MHC class II multimers are still less common; however, recent reports have demonstrated that isolation of extremely rare antigen-specific cells can be facilitated with MHC class II multimer reagents. Furthermore, our protocol can in principle be transferred to any other species, as long as constant region sequences are available. First experiments in the murine system show convincing results (data not shown).

Taken together, with this report we describe the first successful isolation of functional full-length TCR sequences from single T cells. In combination with MHC multimer-based pre-enrichment strategies and combinatorial MHC multimer staining, TCR sequences can be extracted from even extremely rare antigen-specific T cell populations. We believe that this technology will foster TCR identification for clinical applications as well as diagnostics and basic research.

## Materials and Methods

### Reverse Transcription and PCR

Reverse transcription was performed on an AmpliGrid Slide from Advalytix. Single-cells were spotted with the Cyclone system on a MoFlo cell sorter to pre-defined slide positions and were resuspended in 0.5 µl of 50 mM Tris-HCl (pH 8.3), 75 mM KCl, 3 mM MgCl_2_, 1,6 mM dNTPs (Roche), 10 mM DTT (Stratagene), 0.1 mg/ml BSA, 0.1 mg/ml tRNA (Roche), 0.25% Igepal CA-630 (Sigma-Aldrich), 0.8 U/ µl RNAsin Plus (Promega), 1 µM reverse transcription primers each (for sequences see Table S1) and 0.05 rxn/ µl Affinityscript reverse transcriptase. Reverse transcription was performed for 20 min at 51°C followed by 30 min at 70°C on an AmpliSpeed PCR cycler. For primer exonuclease I digest, reaction volume was filled up to 1 µl resulting in a final concentration of 67 mM glycine-KOH (pH 9.5), 6.7 mM MgCl_2_, 10 mM DTT and 1 U/ µl Exonuclease I (Fermentas). Exonuclease-I digest was performed for 30 min at 37°C and enzyme was inactivated for 20 min at 70°C on an AmpliSpeed PCR cycler. For oligo-dG tailing, complete reaction was transferred to a 96 well plate. Tailing was performed in 5 µl of a final concentration of 10 mM MgCl_2,_ 1 mM DTT, 10 mM Tris (pH 7.5) and 2 mM dGTP and 0.75 U/ µl terminal dNTP transferase (Promega), tailing reaction was performed for 45 min at 37°C and enzyme was inactivated for 20 min at 37°C on a Biometra Professional Gradient Cycler. For anchor PCR the reaction volume was filled up to 25 µl with a final concentration of 1× HerculaseII reaction buffer, 0.20 mM dNTPs, 3% formamide, 0.02 rxn/ µl HerculaseII DNA polymerase and 0.5 µM of each primer (for sequences see Table S1). PCR was performed as follows: 94°C 3 min for initial denaturation followed by 24 cycles of 94°C for 15 s, 60°C for 30 s, 72°C for 45 s and 72°C for 5 min as final extension step. 1 µl of product was transferred to nested PCR amplification in separate reactions for α- and β- chain. PCR conditions for nested PCR round I and II were identical to anchor PCR except for primers (for sequences see Table S1).

### Cloning and sequencing

PCR products from single T cell samples were prepared for sequencing by standard blunt end cloning using CloneJet-kit (Fermentas). After ligation, plasmid preparation from transformed DH5α was performed using Pure Yield Mini (Promega). Extracted Plasmids were Sanger sequenced by the commercial provider GATC. Sequencing data was analyzed with Vector NTI Advance and online tools of international ImMunoGeneTics information system for immunoinformatics (IMGT; http://www.imgt.org).

### Retroviral Transduction

PCR products from single-cell PCRs were completed for their truncated constant region by megaprime PCR. In brief, PCR products from α- or β-chain were incubated together with equimolar amounts of PCR product for constant region α- or β- respectively in a PCR mastermix as in the single-cell PCR protocol. Reaction mixture was denatured at 94°C and temperature was reduced from 80°C to 40°C at a rate of 0.1°/s to allow annealing of overlapping sequences. Extension was performed for 45 s at 72°C. This step was repeated three times before addition of primers containing restriction overhangs for subsequent cloning. After addition of primers, 35 cycles of standard PCR were performed. PCR products were cloned into the retroviral expression vector pMP71. For transduction of primary T cells the constant regions of the HER2/neu-specific and WT1-specific TCRα- and β-chain were replaced by codon optimized murine sequences harboring mutations to form an additional cystein bridge. Both TCR chains were combined by a 2A peptide linker sequence (P2A) and cloned into the retroviral vector MP71-PRE. Generation of retroviral particles was performed as previously described [29]. In brief, separate pMP71 expression vectors containing TCRα- and β-chain or a pMP71 vector with a TCR cassette, gag/pol plasmid and amphotropic env plasmid were transfected into HEK 293T cells by calcium phosphate precipitation. Viral supernatant was harvested after 24 h and 48 h and was transferred to Jurkat76 cells and to human PBLs stimulated for two days with plate-bound anti-CD3/CD28 antibodies and 100 UI/ml IL-2 (Proleukin, Novartis, Basel, Switzerland). Transduction of target cells was supported by spin inoculation using retronectin-coated plates (Takara Bio Europe S.A.S., Saint-Germain-en-Laye, France).

### FACS analysis and sorting

FACS antibodies for human cell analysis were CD8 FITC (BD 555366), CD8 PE (BD 555367), CD8 PerCP (BD 345774), CD8 eF450(eBioscience 48-0086-42), CD8 APC (BD 555369), CD3 PE-Cy7 (eBioscience 25-0038-73) and CD19 PE-A610 (Invitrogen MHCD19922). For live/dead discrimination, propidium iodide was used. Samples were analyzed using a CyanLx 9 color flow cytometer (Beckman Coulter). Cell sorting was performed on a MoFlo (Beckman Coulter). FACS data was analyzed with FlowJo v9.5.2 software (Tree Star, Inc.). *Strep*-Tactin PE and *Strep*-Tactin APC were purchased from IBA. Streptavidin BV421 was purchased from Biolegend. Peptide-MHC multimers were generated as described [13], [30]. In brief, MHC heavy chain was urea denatured and refolding in the presence of peptide and beta2 microglobulin was enabled by progressive dialysis of detergent. Complexes were biotinylated by BirA and were purified by FPLC. Monomeric complexes were multimerized by addition of *Strep*-Tactin PE, *Strep*-Tactin APC or Streptavidin BV421. Ratios MHC/backbone and staining dilution were separately titrated for each reagent. HLA-B8 was loaded with CMV IE-1 derived peptides QIKVRVDMV (88-96) and ELRRKMMYM (199-207). The HLA-A2 complex was loaded with Her2/neu derived peptide KIFGSLAFL (369–377) or WT1 specific peptide RMFPNAPYL (126.134) respectively.

### Magnetic cell separation

MACS enrichment with Miltenyi anti-PE nanobeads was performed on Miltenyi MS-columns according to the manufacturers recommendations.

### *In vitro* T cell expansion

T cells were directly FACS sorted into 96 well cell culture plates containing 1 µg/ml Okt3 antibody (Orthoclone), 1 µg/ml anti CD28 (BD 340975), 100 U/ml IL-2 (Proleukin), B-LCLs irradiated with 50 Gy and human PBMCs irradiated with 35 Gy. Cells were kept at 37°C, 95% rel. humidity and 5% CO_2_ in a total volume of 200 µl RPMI^+^, 10% heat inactivated fetal calf serum, Streptomycin, Penicillin, Gentamycin.

### Cells and cell lines

Blood was taken from healthy donors. Written informed consent was obtained from the donors, and usage of the blood samples was approved according to national law by the local Institutional Review Board (Ethikkommission der Medizinischen Fakultät der Technischen Universität München). PBMCs were extracted from whole blood, pre-diluted 1∶1 with PBS (pH 7.4), by density gradient centrifugation for 20 min at 2000 rpm on a layer of Ficoll.

Jurkat 76 cells were grown at 37°C, 95% humidity, 5% CO_2_ in 1640 RPMI medium supplemented with 10% fetal calf serum and 100 U/ml penicillin, 100 U/ml streptomycin and gentamycin.

The HER2^+^/HLA-A2^−^overian cancer cell line SKOV-3 (American Type Culture Collection (ATCC), Manassas, VA, USA: HTB-77), the HLA-A2^+^transfected cell line SKOV-3-A2 (supplied by M. L. Disis; University of Washington, Seattle, WA, USA) and the HEK 293T cell line (ATCC: CRL-11268) were cultured in DMEM medium with Glutamax I (Life Technologies, Darmstadt, Germany) supplemented with 10% heat-inactivated fetal calf serum (inact. FCS; Biochrom AG, Berlin, Germany) and 100 IU/ml of penicillin/streptomycin (P/S; Life Technologies). T cell medium (TCM) consisting of RPMI 1640 medium with Glutamax I (Life Technologies) supplemented with 10% inact. FCS (Pan Biotech, Aidenbach, Germany), 10 mM HEPES, and 100 IU/ml of P/S was used to culture T2 cells (ATCC: CRL- 1992) and primary T cells.

### Functional analysis of T cells

TCR-modified PBLs were analyzed in chromium (^51^Cr) release assays and interferon (IFNγ)- secretion was measured by enzyme-linked immunosorbent assays (ELISA). To determine cytolytic activity 2×10^3^ peptide-loaded T2 cells (1 µM) labeled with ^51^Cr were incubated for 4 h with TCR-modified PBLs at different effector/target ratios (E/T ratios). T2 cells were pulsed with HER2_369–377_: KIFGSLAFL and WT-1_126–134_: RMFPNAPYL peptides (Biosynthan, Berlin, Germany). Spontaneous release was determined using T2 cells incubated without T cells and the transfer of T2 cells directly to the solid scintillator-coated lumaplates without incubation produced maximal release values. All samples were run in duplicates and the specific lysis was calculated as follows:% specific cytotoxicity  =  [mean sample release (cpm) - mean spontaneous release (cpm)]/[mean maximal release (cpm) - mean spontaneous release (cpm)] ×100. For determination of cytokine release IFNγ was measured after coculture of 1×10^5^ TCR-modified PBLs with the same number of T2 cells pulsed with titrated amounts of peptide or with tumor cells for 24 h by ELISA (BD Biosciences, Heidelberg, Germany) according to the manufacturer's instructions. Samples were run in triplicates.

## Supporting Information

Figure S1 **Overview of the TCR-SCAN priming strategy exemplified for α-chain.** For each chain three serially arranged reverse-transcription-primers (TCRAC 1/2/3) were applied. After exonuclease digest and 3′addition of oligo-dG the next PCR step (anchor PCR) was performed. Forward primer (dC-anchor-PTO) bound to the oligo-dG stretch and reverse primer (TCRAC 4) bound to the TCR constant region. Two subsequent rounds of nested PCR were performed with forward primers (Adaptor 1and Adaptor 2) binding to the artificial priming site that was extended in the previous step. Reverse primers in nested PCR I and II (TCRAC 5 and TCRAC 6) bound in the constant region. Priming for β-chain was analogous.
(TIF)

Figure S2 **10 T cells per sample were isolated, and TCR α-β-chains were amplified by RACE-PCR as described in** **Figure 1** **.** Reverse transcription temperature was varied between samples as indicated.
(TIF)

Figure S3 **CDR3 sequences from four closely related TCR sequences with the same length as shown in** **Figure 3E** **were compared by multiple sequence alignment.** Grade of identity and similarity was calculated by matrix blosum62mt2 under Vector NTI.
(TIF)

Figure S4 **(A) PBMCs from a healthy donor were labeled with MHC multimer A2/WT1_126–134_ and were enriched by magnetic cell separation.** Left FACS-plot shows cell fraction after enrichment with the MHC multimer backbone and serves as purity control. Right FACS plot shows cells after enrichment with functional MHC multimer. (B) Single cells were sorted for TCR-SCAN and PCR-products were sequenced. Table shows characteristics of two TCRs from this experiment. TCR5A was identified once TCR5B three times. (C) TCR5A and TCR5B were transduced to human PBMCs and MHC-multimer staining was performed. FACS plots show living lymphocytes after transduction.
(TIF)

Table S1 **All primers that were used in the single cell PCR protocol are described.** The column step indicates where this primer was used. In addition we show the name we used and provide the nucleotide sequences.
(TIF)

Table S2 **All nucleotide sequences of the α-chain rearrangements of CMV specific TCRs shown in** **Figures 2** **and** **3** **are summarized.** The corresponding sequence can be matched to the information in the respective figures by the label. We have subdivided the nucleotide sequences into the different domaints i.e. V- and J segment and the additional non-germline sequences that have been inserted during somatic recombination (P- and N- nucleotides).
(TIF)

Table S3 **All nucleotide sequences of the α-chain rearrangements of CMV specific TCRs shown in** **Figures 2** **and** **3** **are summarized.** The corresponding sequence can be matched to the information in the respective figures by the label. We have subdivided the nucleotide sequences into the different domaints i.e. V-, D- and J-segment and the additional non-germline sequences that have been inserted during somatic recombination (P- and N- nucleotides).
(TIF)
