# Supplementary figures and images for: MHC Multimer-Guided and Cell Culture-Independent Isolation of Functional T Cell Receptors from Single Cells Facilitates TCR Identification for Immunotherapy
Source: PLoS One. 2013 Apr 26;8(4):e61384. doi: 10.1371/journal.pone.0061384 (PMC3637308; doi:10.1371/journal.pone.0061384)

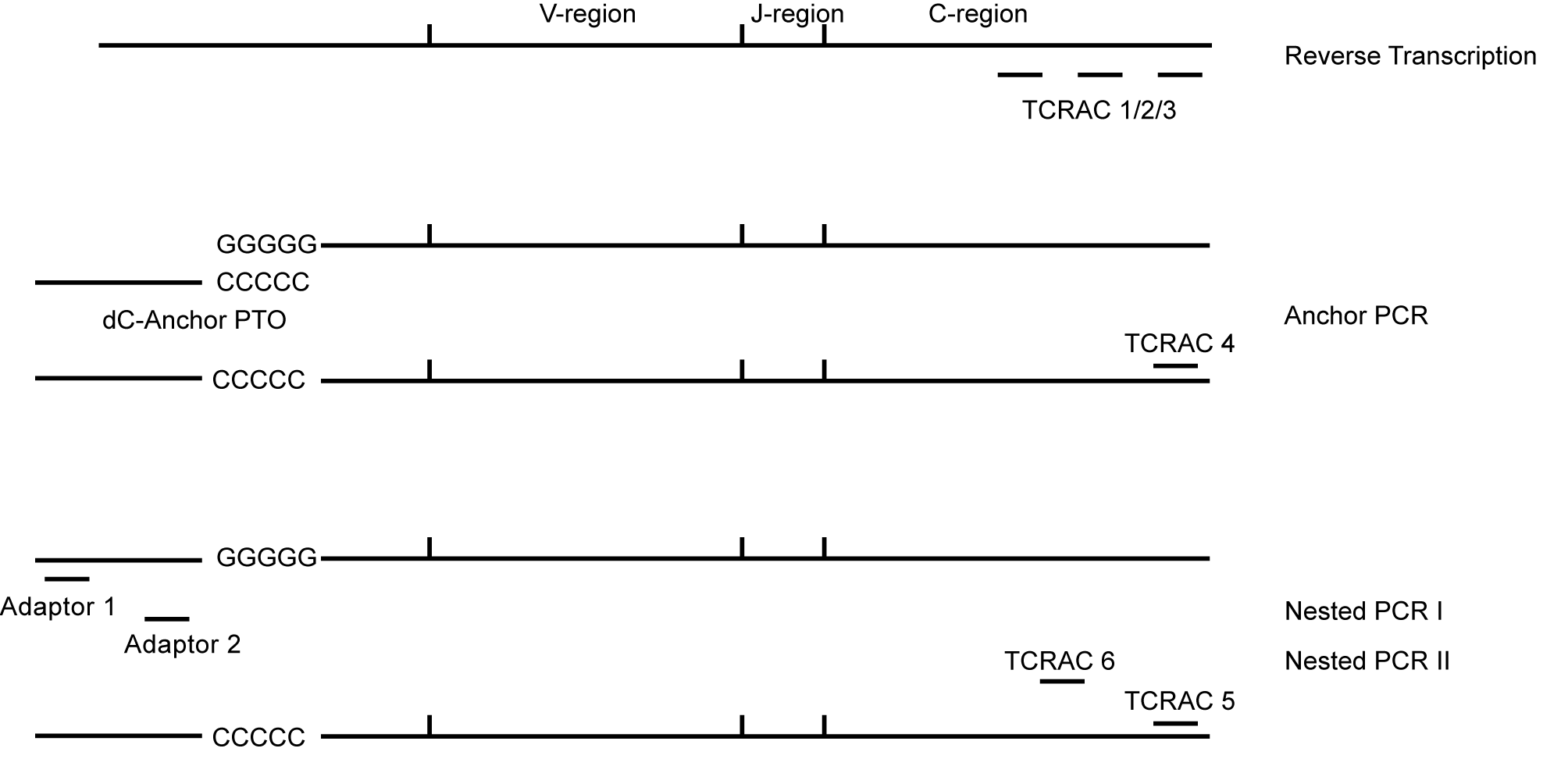

Supplement: Figure S1 — Overview of the TCR-SCAN priming strategy exemplified for α-chain. For each chain three serially arranged reverse-transcription-primers (TCRAC 1/2/3) were applied. After exonuclease digest and 3′addition of oligo-dG the next PCR step (anchor PCR) was performed. Forward primer (dC-anchor-PTO) bound to the oligo-dG stretch and reverse primer (TCRAC 4) bound to the TCR constant region. Two subsequent rounds of nested PCR were performed with forward primers (Adaptor 1and Adaptor 2) binding to the artificial priming site that was extended in the previous step. Reverse primers in nested PCR I and II (TCRAC 5 and TCRAC 6) bound in the constant region. Priming for β-chain was analogous. (TIF) [file pone.0061384.s001.tif]

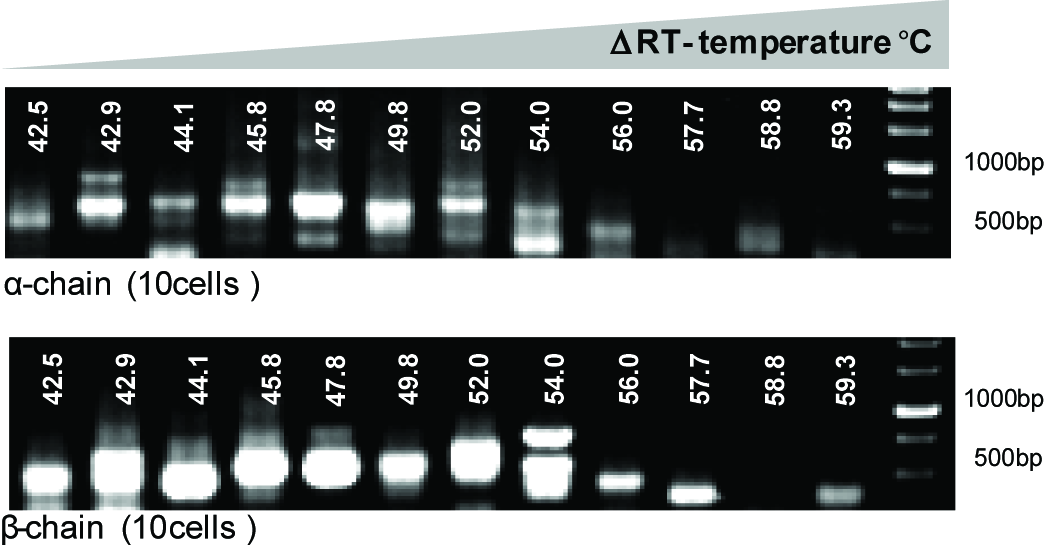

Supplement: Figure S2 — 10 T cells per sample were isolated, and TCR α-β-chains were amplified by RACE-PCR as described in Figure 1 . Reverse transcription temperature was varied between samples as indicated. (TIF) [file pone.0061384.s002.tif]

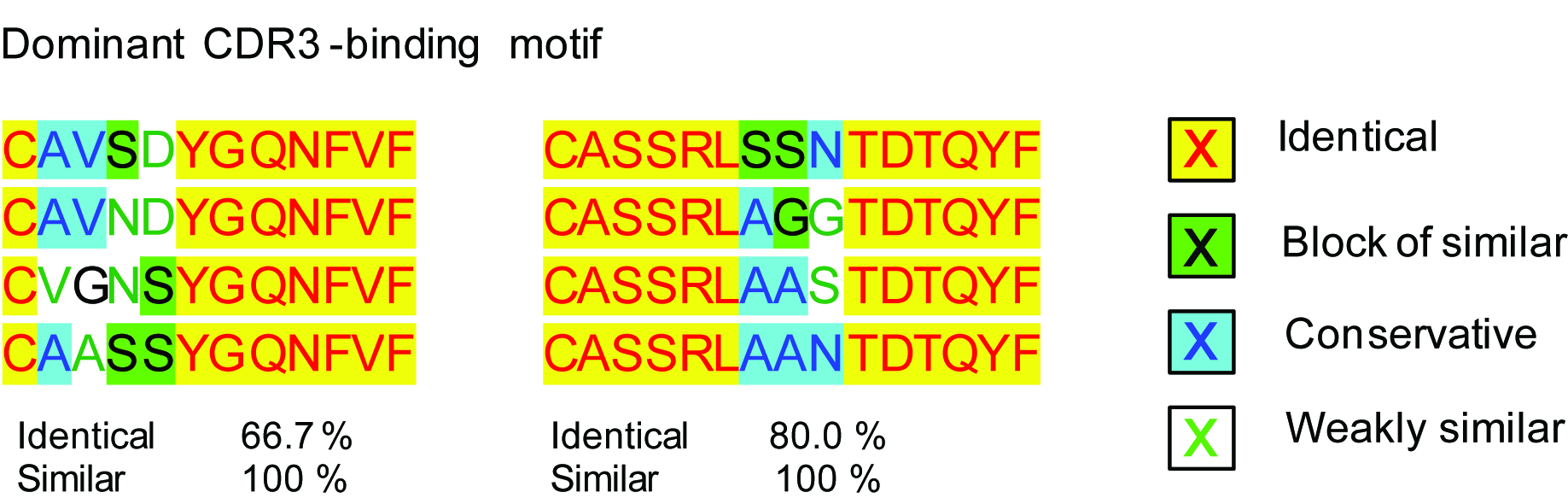

Supplement: Figure S3 — CDR3 sequences from four closely related TCR sequences with the same length as shown in Figure 3E were compared by multiple sequence alignment. Grade of identity and similarity was calculated by matrix blosum62mt2 under Vector NTI. (TIF) [file pone.0061384.s003.tif]

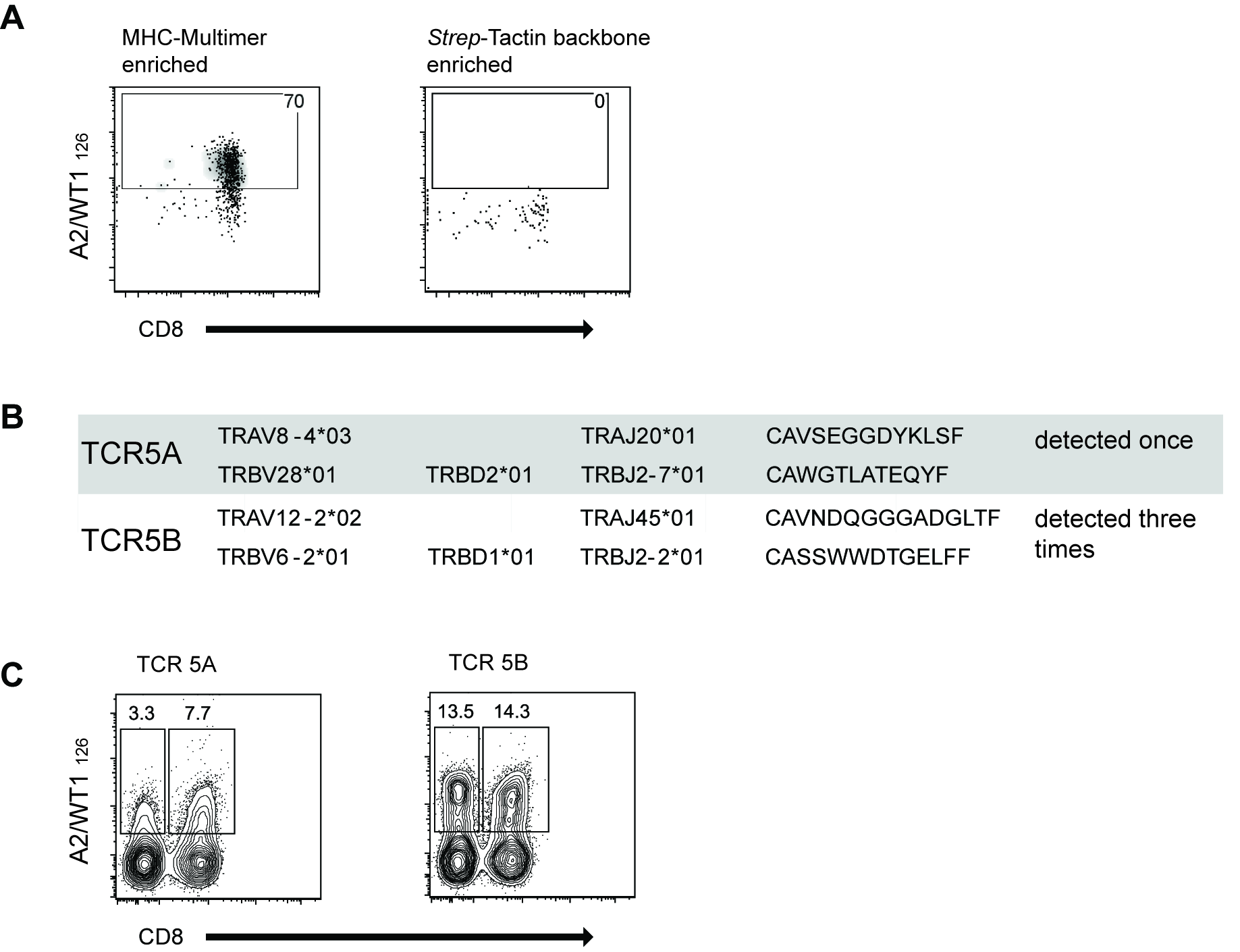

Supplement: Figure S4 — (A) PBMCs from a healthy donor were labeled with MHC multimer A2/WT1126–134 and were enriched by magnetic cell separation. Left FACS-plot shows cell fraction after enrichment with the MHC multimer backbone and serves as purity control. Right FACS plot shows cells after enrichment with functional MHC multimer. (B) Single cells were sorted for TCR-SCAN and PCR-products were sequenced. Table shows characteristics of two TCRs from this experiment. TCR5A was identified once TCR5B three times. (C) TCR5A and TCR5B were transduced to human PBMCs and MHC-multimer staining was performed. FACS plots show living lymphocytes after transduction. (TIF) [file pone.0061384.s004.tif]

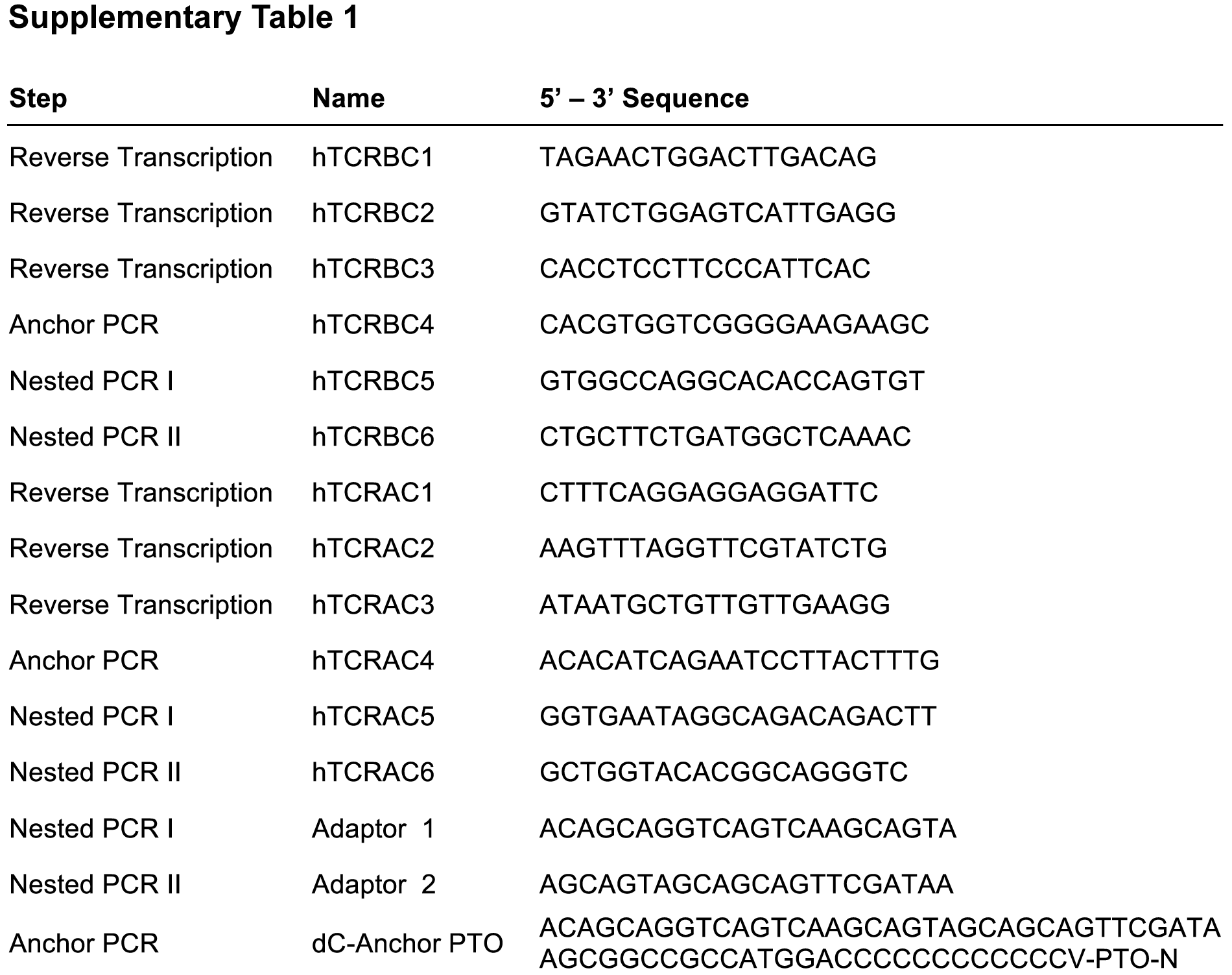

Supplement: Table S1 — All primers that were used in the single cell PCR protocol are described. The column step indicates where this primer was used. In addition we show the name we used and provide the nucleotide sequences. (TIF) [file pone.0061384.s005.tif]

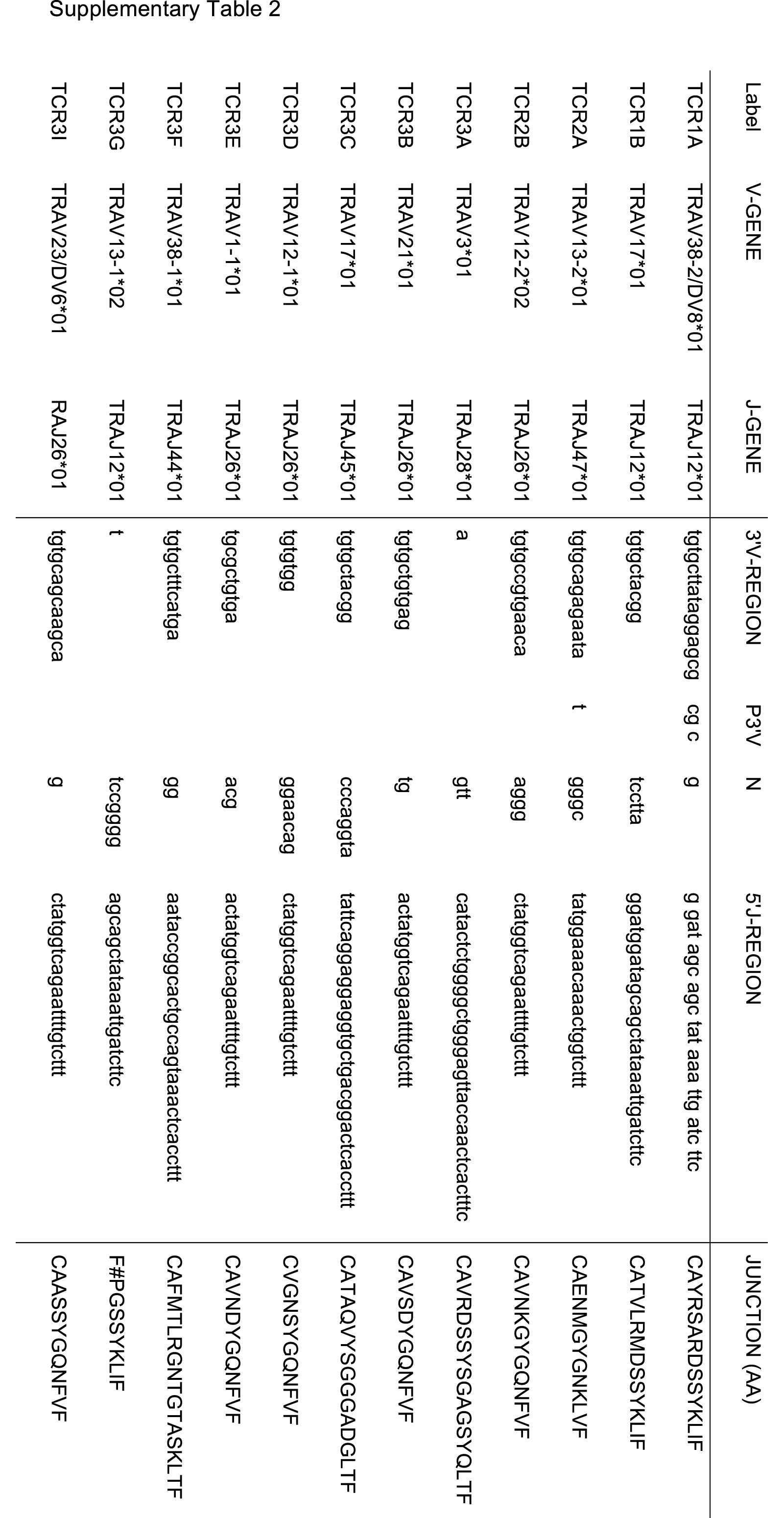

Supplement: Table S2 — All nucleotide sequences of the α-chain rearrangements of CMV specific TCRs shown in Figures 2 and 3 are summarized. The corresponding sequence can be matched to the information in the respective figures by the label. We have subdivided the nucleotide sequences into the different domaints i.e. V- and J segment and the additional non-germline sequences that have been inserted during somatic recombination (P- and N- nucleotides). (TIF) [file pone.0061384.s006.tif]

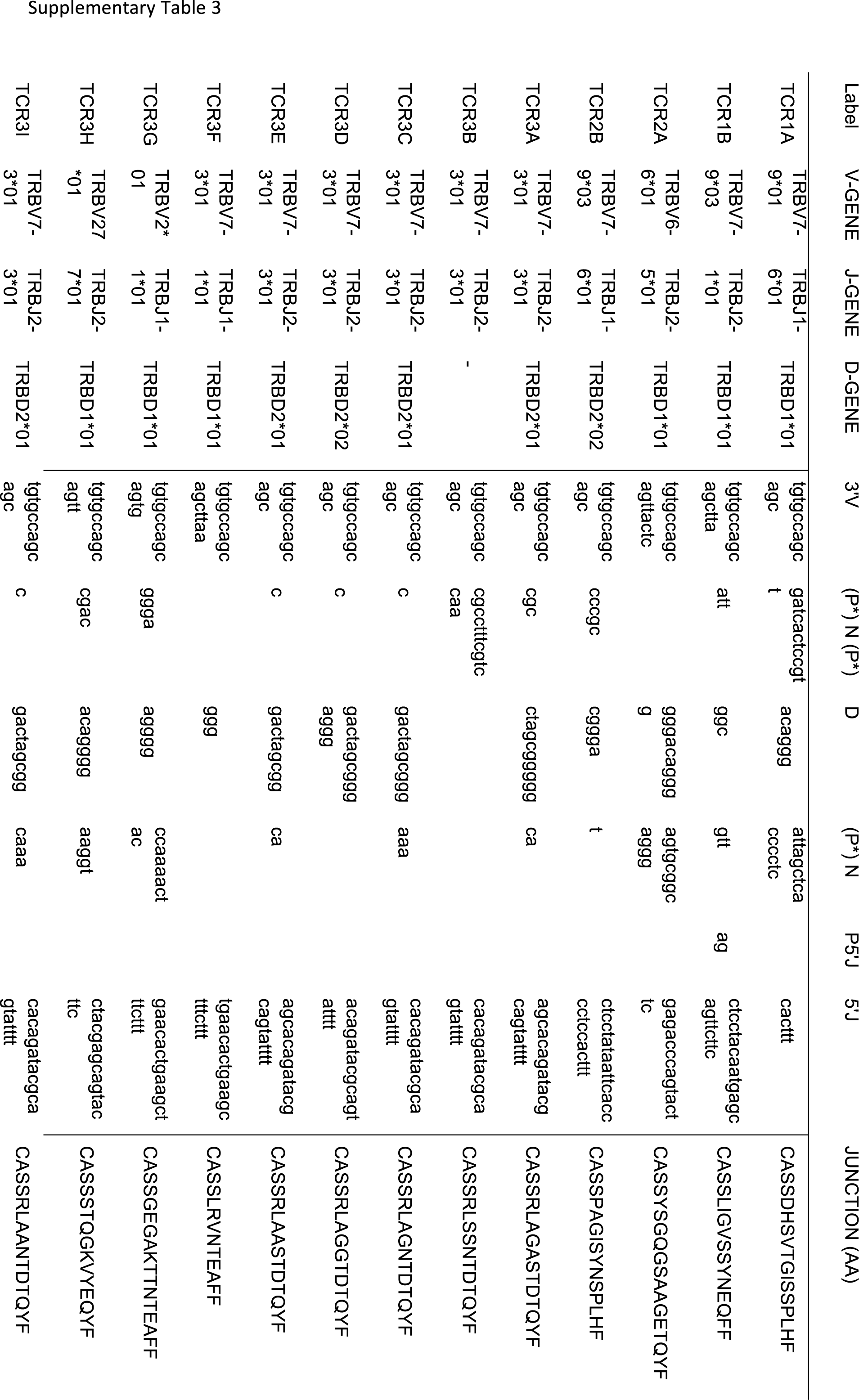

Supplement: Table S3 — All nucleotide sequences of the α-chain rearrangements of CMV specific TCRs shown in Figures 2 and 3 are summarized. The corresponding sequence can be matched to the information in the respective figures by the label. We have subdivided the nucleotide sequences into the different domaints i.e. V-, D- and J-segment and the additional non-germline sequences that have been inserted during somatic recombination (P- and N- nucleotides). (TIF) [file pone.0061384.s007.tif]
